# ASO Author Reflections: Increasing Time of Diagnosis to Breast Cancer Surgery

**DOI:** 10.1245/s10434-024-15295-9

**Published:** 2024-04-29

**Authors:** Cary Kaufman, Terry Sarantou, Cory Donovan, Ayfer Kamali Polat, Peggy Thomas, Bonnielyn Mack

**Affiliations:** 1 Department of Surgery, Bellingham Regional Breast Center, University of Washington, Bellingham, WA USA; 2 Atrium Health, General Surgery, Charlotte, NC USA; 3 Legacy Good Samaritan Medical Center, Surgical Oncology, Legacy Health System, Portland, OR USA; 4 Department of General Surgery, Ondokuz Mayis University, Samsun, Turkey; 5 Penrose-St Francis Health Services, Oncology, Colorado Springs, CO USA; 6 Breast Center, Portsmouth Regional Hospital, Portsmouth, NH USA

## Past

We appreciate the multiple authors who have demonstrated that increasing time to surgery (TTS) is associated with decreases in survival.^1^ Prior focus on TTS gave attention to patient and tumor characteristics and less on the clinical facility where the diagnosis and initial treatment occurred. Our prior article demonstrated that improvements have occurred in time to diagnostic imaging and time to core needle biopsy diagnosis with relationships to clinical facility characteristics.^2^ Average time from diagnosis to surgery (ATTS) has increased by 28% (*p* < 0.001) over 15 years (2005–2019) [personal communication, April 2024]. Some characteristics of the facilities appear to relate to delays in ATTS.

## Present

Today there are more demands on the preoperative assessment of patients, including use of breast magnetic resonance images (MRIs), genetic testing, second opinions, reconstruction options, insurance approvals, and others. Nonetheless, some centers perform much better than others. Variables identifying well-performing centers include geographic location, population density, mammogram volume, cancer volume, services provided, and facility ownership. Many centers may not calculate their own TTS or ATTS. If survival is impacted by delays in TTS, then awareness of one’s own TTS/ATTS is important.

## Future

This effort has shown that prolongation of ATTS has occurred in all types of facilities over the last 15 years (pre-pandemic). Since increasing TTS is associated with a measurable decrease in survival, our burden is to focus on causes of prolongation in our systems of care. Efforts should be directed towards routine assessment and reporting of TTS/ATTS with the goal of identifying correctable issues. Tracking of each facility’s processes of care, how samples are identified, processed, and communicated, referrals made, preoperative assessment occurred, and time of surgery performed will be necessary as we approach existing increases in TTS/ATTS. The sooner facilities tackle this problem of prolonged TTS/ATTS, the sooner our patients will benefit from their efforts.

## References

[1] Bleicher RJ, Ruth K, Sigurdson ER (2016). Time to Surgery and Breast Cancer Survival in the United States. JAMA Oncol..

[2] Donovan CA, Kaufman CS, Thomas KA (2023). Timeliness of Breast Diagnostic Imaging and Biopsy in Practice: 15 Years of Collecting, Comparing, and Defining Quality Breast Cancer Care. Ann Surg Oncol..

